# Whole-Genome Sequences of *Salmonella* Isolates from an Ecological Wastewater Treatment System

**DOI:** 10.1128/MRA.00456-20

**Published:** 2020-06-04

**Authors:** Charles J. Connolly, Laura Kaminsky, Gabriella N. Pinto, Priscilla C. Sinclair, Gyasu Bajracharya, Runan Yan, Erin M. Nawrocki, Edward G. Dudley, Jasna Kovac

**Affiliations:** a Foodborne Pathogens Genomic Epidemiology Course, Department of Food Science, The Pennsylvania State University, University Park, Pennsylvania, USA; b Department of Food Science, The Pennsylvania State University, University Park, Pennsylvania, USA; University of Arizona

## Abstract

Twenty-seven *Salmonella* isolates were collected from four locations within an ecological wastewater treatment system located at The Pennsylvania State University and were subjected to whole-genome sequencing. The sequences obtained were used for *in silico* characterization, including serotyping and phylogenetic relatedness analysis.

## ANNOUNCEMENT

Twenty-seven *Salmonella* isolates were isolated from wastewater samples collected from four locations (wastewater delivery truck, anoxic tank, clarifier, and pond) within an experimental ecological wastewater treatment system located at The Pennsylvania State University. Samples were collected on four dates (31 May, 6 June, 21 June, and 2 July) in 2019. The collected wastewater samples were centrifuged, filtered, and enriched for *Salmonella* spp. following the Food and Drug Administration’s Bacteriological Analytical Manual protocol (1). Presumptive *Salmonella* colonies isolated from selective differential agars were subcultured in brain heart infusion (BHI) broth at 35°C for 24 h and were used for DNA extraction with DNeasy blood and tissue kits (Qiagen). Extracted DNA was used to prepare Illumina Nextera XT libraries, which were sequenced on an Illumina MiSeq system with 300-bp paired-end reads, using a 600-cycle v3 kit.

Read quality was assessed with FastQC v0.11.5 (2) using default parameters. Low-quality bases and adapters were removed with Trimmomatic v0.36 (3) using the parameters recommended in the program manual. Trimmed reads were assembled *de novo* with SPAdes v3.14.0 (4), using k-mer lengths of 99 and 127 and a “careful” option. Assembly qualities were assessed with QUAST 4.6.1 (5). Average coverage was calculated using BWA v0.7.12 (6) and SAMtools v1.5 (7). Sequencing and SPAdes assembly metrics are shown in Table 1. The assemblies available in the NCBI nucleotide database were generated prior to this study using SKESA v2.2 (8) and were annotated using the NCBI Prokaryotic Genome Annotation Pipeline (9), but they were not used in this study. Serotypes were identified using sistr_cmd v1.0.2 (10). Core single-nucleotide polymorphisms (SNPs) for all 27 isolates were identified using kSNP3 v3.1.2 (11) with a k-mer size of 19 and were used to construct a maximum likelihood tree in RAxML v8.2, using a GTR-GAMMA model with Lewis correction and 1,000 bootstrap iterations (12). Another tree, containing only isolates of *Salmonella enterica* serotype Montevideo, was constructed using core genome SNPs identified with the CFSAN SNP pipeline Galaxy v1.0.1 (13), using accession number SRR9853881 as the reference genome. The cytolethal distending toxin-encoding gene *cdtB* (NP_456275) was added to the BTyper 2.3.1 virulence database and searched for using default parameters (14). BTyper was also used for the detection of antimicrobial resistance genes, with default parameters.

**TABLE 1:** Sequencing metrics for the 27 *Salmonella* whole-genome sequences and assemblies

SRA accession no.	Total no. of contigs	No. of contigs ≥1,000 bp	Assembly length (bp)	*N*_50_ (bp)	Avg coverage (×)	GC content (%)	No. of reads
SRR9853520	121	56	4,974,711	216,902	85	52.14	1,802,482
SRR9853529	83	37	4,648,670	272,202	107	52.19	2,173,272
SRR9853530	74	31	4,646,343	362,580	68	52.19	1,340,334
SRR9853590	114	78	4,643,063	108,252	58	52.20	1,136,098
SRR9853876	93	59	4,642,802	149,195	55	52.19	1,104,466
SRR9853881	71	33	4,645,578	151,198	134	52.20	2,992,046
SRR9853882	71	34	4,643,296	229,853	40	52.19	789,878
SRR9853887	79	41	4,644,479	225,328	64	52.19	1,265,342
SRR9853888	127	68	4,679,950	110,361	65	52.16	1,386,824
SRR9853890	75	33	4,646,670	272,047	92	52.19	1,840,696
SRR9853891	73	31	4,646,815	272,202	58	52.19	1,151,244
SRR9853906	150	102	4,646,133	83,121	40	52.19	878,332
SRR9854047	73	24	4,675,646	426,302	43	52.17	859,994
SRR9854048	83	43	4,644,127	225,703	80	52.20	1,565,354
SRR9854070	79	39	4,646,207	225,328	131	52.19	2,835,590
SRR9854072	75	31	4,648,893	272,202	72	52.19	1,431,874
SRR9854075	84	45	4,645,124	221,805	72	52.19	1,459,050
SRR9854076	77	36	4,614,194	225,327	94	52.19	2,087,984
SRR9854078	100	61	4,639,441	140,737	117	52.20	2,277,850
SRR9854079	73	36	4,671,031	144,819	60	52.21	1,195,606
SRR9854081	79	36	4,646,637	227,152	99	52.19	2,167,306
SRR9854098	96	58	4,672,037	147,739	71	52.20	1,412,384
SRR9854100	94	42	4,652,181	229,931	76	52.18	1,557,636
SRR9854104	74	28	4,717,129	418,892	65	52.24	1,319,400
SRR9854255	76	38	4,644,689	225,703	59	52.19	1,218,688
SRR9943133	87	41	4,644,304	261,568	36	52.20	711,564
SRR9943578	158	111	4,637,285	72,494	26	52.22	566,540

**FIG 1:**
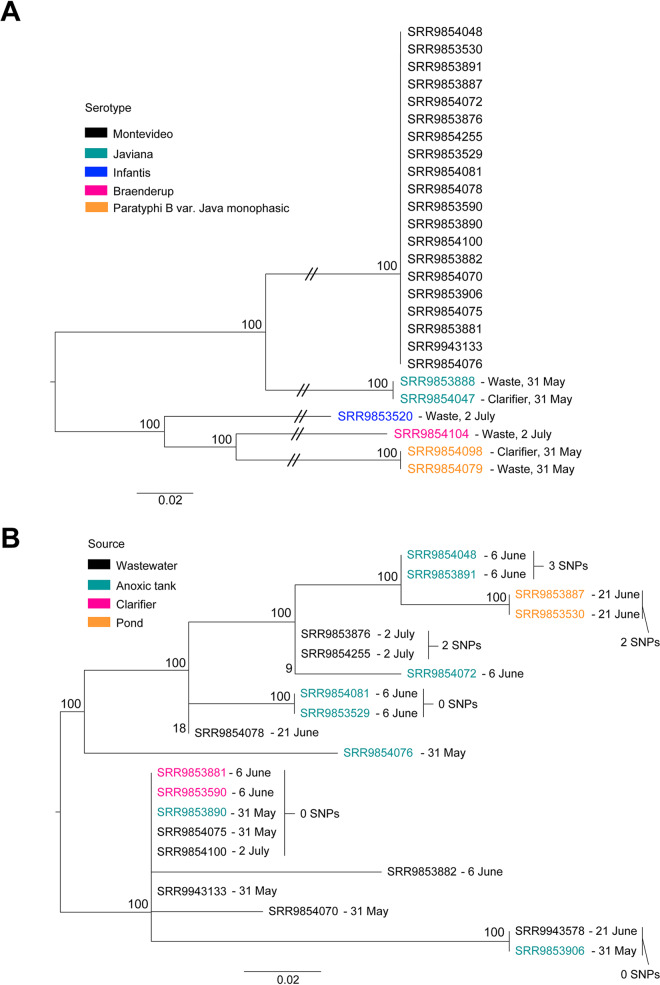
Phylogenetic diversity of *Salmonella* isolates collected from an experimental ecological wastewater treatment system. (A) Maximum likelihood tree of all isolates, built using core genome SNPs identified with kSNP3. Isolate accession numbers are colored according to the serotype identified using sistr_cmd v1.0.2 and are labeled with the sampling date and location. (B) Maximum likelihood tree of *Salmonella* Montevideo isolates, built using core genome SNPs identified with the CFSAN SNP pipeline Galaxy v1.0.1. Isolate SRR9853881 was used as the reference genome. Isolate accession numbers are colored according to the sampling location and are labeled with the sampling date. Bootstrap values below 60 are not displayed.

The average coverage, number of contigs larger than 1,000 bp, GC content, and *N*_50_ value for the assemblies obtained were 68.84×, 47.11, 52.19%, and 217,735 bp, respectively. Isolates were identified as *S. enterica* serotypes Montevideo (*n* = 21), Javiana (*n* = 2), Infantis (*n* = 1), Paratyphi B (*n* = 2), and Braenderup (*n* = 1). Untreated wastewater samples housed the greatest diversity of serotypes, as they had all five serotypes detected. Only *S*. Montevideo isolates were detected in samples from the anoxic tank and pond (Fig. 1A and B), while *S*. Montevideo, *S*. Javiana, and *S*. Paratyphi B were detected in samples from the clarifier. Among isolates of *S*. Montevideo, four pairs of isolates obtained from the same location on the same day had nearly identical core genomes (Fig. 1B). There was also a cluster of highly similar isolates spanning several sampling dates and locations (Fig. 1B), suggesting potential persistence of a specific genotype within the wastewater treatment system during the sampling period. The *cdtB* gene (15) was detected in all isolates of *S*. Montevideo and *S*. Javiana. Nine antimicrobial resistance genes were also detected, i.e., *aac6-ly* (*n* = 26), *fosA7* (*n* = 21), *qnrD* (*n* = 2), *aac6-laa*, *aadA1-pm*, *sul1*, *sul2*, *tetA*, and *tetR* (*n* = 1).

### Data availability.

The sequencing reads were deposited in the NCBI database under BioProject number PRJNA357723. The SRA accession numbers are SRR9854075, SRR9853529, SRR9853882, SRR9853520, SRR9854070, SRR9854072, SRR9854081, SRR9853906, SRR9854255, SRR9853590, SRR9943578, SRR9853887, SRR9853530, SRR9853876, SRR9853890, SRR9854100, SRR9854047, SRR9854104, SRR9853888, SRR9853891, SRR9854079, SRR9854076, SRR9854098, SRR9854078, SRR9853881, SRR9943133, and SRR9854048.

## References

[1] Andrews WH, Wang H, Jacobson A, Hammack T 2018 Chapter 5. Salmonella *In* Bacteriological analytical manual. Food and Drug Administration, Silver Spring, MD https://www.fda.gov/food/foodscienceresearch/laboratorymethods/ucm070149.htm.

[2] Trivedi UH, Cézard T, Bridgett S, Montazam A, Nichols J, Blaxter M, Gharbi K 2014 Quality control of next-generation sequencing data without a reference. Front Genet 5:111. doi:10.3389/fgene.2014.00111. 24834071 PMC4018527

[3] Bolger AM, Lohse M, Usadel B 2014 Trimmomatic: a flexible trimmer for Illumina sequence data. Bioinformatics 30:2114–2120. doi:10.1093/bioinformatics/btu170. 24695404 PMC4103590

[4] Bankevich A, Nurk S, Antipov D, Gurevich AA, Dvorkin M, Kulikov AS, Lesin VM, Nikolenko SI, Pham S, Prjibelski AD, Pyshkin AV, Sirotkin AV, Vyahhi N, Tesler G, Alekseyev MA, Pevzner PA 2012 SPAdes: a new genome assembly algorithm and its applications to single-cell sequencing. J Comput Biol 19:455–477. doi:10.1089/cmb.2012.0021. 22506599 PMC3342519

[5] Gurevich A, Saveliev V, Vyahhi N, Tesler G 2013 QUAST: quality assessment tool for genome assemblies. Bioinformatics 29:1072–1075. doi:10.1093/bioinformatics/btt086. 23422339 PMC3624806

[6] Li H, Durbin R 2009 Fast and accurate short read alignment with Burrows-Wheeler transform. Bioinformatics 25:1754–1760. doi:10.1093/bioinformatics/btp324. 19451168 PMC2705234

[7] Li H, 1000 Genome Project Data Processing Subgroup, Handsaker B, Wysoker A, Fennell T, Ruan J, Homer N, Marth G, Abecasis G, Durbin R 2009 The Sequence Alignment/Map format and SAMtools. Bioinformatics 25:2078–2079. doi:10.1093/bioinformatics/btp352. 19505943 PMC2723002

[8] Souvorov A, Agarwala R, Lipman DJ 2018 SKESA: strategic k-mer extension for scrupulous assemblies. Genome Biol 19:153. doi:10.1186/s13059-018-1540-z. 30286803 PMC6172800

[9] Tatusova T, DiCuccio M, Badretdin A, Chetvernin V, Nawrocki EP, Zaslavsky L, Lomsadze A, Pruitt KD, Borodovsky M, Ostell J 2016 NCBI Prokaryotic Genome Annotation Pipeline. Nucleic Acids Res 44:6614–6624. doi:10.1093/nar/gkw569. 27342282 PMC5001611

[10] Yoshida CE, Kruczkiewicz P, Laing CR, Lingohr EJ, Gannon VP, Nash JH, Taboada EN 2016 The *Salmonella* In Silico Typing Resource (SISTR): an open Web-accessible tool for rapidly typing and subtyping draft *Salmonella* genome assemblies. PLoS One 11:e0147101. doi:10.1371/journal.pone.0147101. 26800248 PMC4723315

[11] Gardner SN, Slezak T, Hall BG 2015 kSNP3.0: SNP detection and phylogenetic analysis of genomes without genome alignment or reference genome. Bioinformatics 31:2877–2878. doi:10.1093/bioinformatics/btv271. 25913206

[12] Stamatakis A 2014 RAxML version 8: a tool for phylogenetic analysis and post-analysis of large phylogenies. Bioinformatics 30:1312–1313. doi:10.1093/bioinformatics/btu033. 24451623 PMC3998144

[13] Davis S, Pettengill JB, Luo Y, Payne J, Shpuntoff A, Rand H, Strain E 2015 CFSAN SNP Pipeline: an automated method for constructing SNP matrices from next-generation sequence data. PeerJ Comput Sci 1:e20. doi:10.7717/peerj-cs.20.

[14] Carroll LM, Kovac J, Miller RA, Wiedmann M 2017 Rapid, high-throughput identification of anthrax-causing and emetic *Bacillus cereus* group genome assemblies via BTyper, a computational tool for virulence-based classification of *Bacillus cereus* group isolates by using nucleotide sequencing data. Appl Environ Microbiol 83:e01096-17. doi:10.1128/AEM.01096-17. 28625989 PMC5561296

[15] Miller RA, Wiedmann M 2016 Dynamic duo—the *Salmonella* cytolethal distending toxin combines ADP-ribosyltransferase and nuclease activities in a novel form of the cytolethal distending toxin. Toxins (Basel) 8:121. doi:10.3390/toxins8050121. PMC4885037 27120620

